# Cultural adaptation of digital healthcare tools: a cross-sectional survey of caregivers and patients

**DOI:** 10.1186/s41256-025-00439-5

**Published:** 2025-08-21

**Authors:** Shuya Zhou, Muzi Shen, Xinge Tao, Shasha Han

**Affiliations:** 1 School of Population Medicine and Public Health, Chinese Academy of Medical Sciences and Peking Union Medical College, Beijing, People’s Republic of China; 2 State Key Laboratory of Respiratory Health and Multimorbidity, Beijing, People’s Republic of China; 3 Key Laboratory of Pathogen Infection Prevention and Control (Peking Union Medical College), Ministry of Education, Beijing, People’s Republic of China

## Abstract

**Background:**

Optimizing the cultural accessibility of digital healthcare tools requires understanding user perspectives on usability features and cultural appropriateness.

**Methods:**

A cross-sectional survey of 3,030 caregivers (mean age 44.0, 52.9% female) and 2,108 inpatients (mean age 49.7, 54.0% female) at a Guangxi medical center (July–October 2024) assessed experiences with digital tools, support needs, and preferences for culturally adaptive features.

**Results:**

Caregivers reported a higher adoption of digital tools than patients (caregivers: 87.1% vs. patients: 62.0%, *P*-value < .001), yet 81.1% of caregivers reported unmet needs. Both groups (caregivers: 67.0%; patients: 64.0%) prioritized integrating traditional medicine over other cultural factors (language diversity, traditional medicine, folk customs, and medical resource availability). Caregivers valued interactive health management tools (73.3% vs. 66.7% among patients, *P*-value < .001) and user feedback mechanisms (61.2% vs. 55.0% among patients, *P*-value < .001) more than patients.

**Conclusions:**

Despite high adoption, caregivers report significant gaps in culturally relevant support. Digital health interventions should prioritize user-centered designs, incorporating traditional medicine and addressing the divergent preferences of caregivers and patients.

## Introduction

Digital healthcare technologies offer a promising avenue for empowering caregivers by providing access to information, training resources, and support networks. However, successful adoption and utilization of digital healthcare tools depend on understanding the unique needs and preferences of caregivers, the distinct needs between them and patients, and the socio-cultural context in which they operate [1], particularly in regions with blending modern and traditional medical practices [2–5]. The Guangxi Zhuang Autonomous Region, with its rich ethnolinguistic diversity (e.g., Zhuang and Yao minorities) and prevalent traditional medicine, offers an ideal setting to study cultural adaptation in healthcare. This resource-limited area also serves as a crucial microcosm for global health equity, where culturally tailored digital innovations could help to address caregiving disparities [6, 7]. Our study aimed to investigate caregiver and patient perspectives on features that could enhance the usability and cultural appropriateness of digital healthcare tools.

## Methods

We conducted a cross-sectional survey involving 3424 caregivers and 2678 inpatients they cared for between July 8 and October 31, 2024. The survey was conducted at a large medical center (The First Affiliated Hospital of Guilin Medical University) in the Guangxi Zhuang Autonomous Region, which is one of the five autonomous regions in China and is home to the Zhuang and Yao ethnic populations. We used convenience sampling, selecting participants based on their availability and accessibility. Inclusion criteria for this study were: 1) hospitalized inpatients admitted for at least 48 h and their primary caregivers; 2) residency within the Guangxi Zhuang Autonomous Region; and 3) provision of informed consent. Participants were excluded if they presented with severe cognitive impairment, severe psychological disorders, or any other condition precluding the completion of questionnaires. The internal consistency of the survey questionnaires was robust (Cronbach’s α > 0.70 for all dimensions), and face validity was established through iterative feedback from 30 patients and caregivers in the pilot study conducted in June 2024, ensuring relevance to the inpatient contexts. Continuous variables were summarized using means and standard deviations, while categorical variables were reported as proportions and percentages. The chi-squared test was used to compare the distribution of categorical variables between caregivers and patients. The Rao-Scott correction was applied to multiple-response questionnaires. Descriptive statistical analyses were conducted from November 15, 2024, to February 25, 2025, using R version 4.2.2.

Approval for the study was granted by the research ethics board of the Chinese Academy of Medical Sciences & Peking Union Medical College (CAMS&PUMC-IEC-2023-017). Participant consent was obtained before the survey. We adhered to the Strengthening the Reporting of Observational Studies in Epidemiology (STROBE) guidelines for cross-sectional studies.

## Results

Characteristics of the 3030 caregivers (mean [SD] age, 44.0 [12] years; 1603 [52.9%] female) and 2108 inpatients they cared for (mean [SD] age, 49.7 [17.1] years; 1139 [54.0%] female) who completed the survey (response rate, caregivers: 88.5%; inpatients: 78.7%) are detailed in Table 1. Most caregivers self-reported their race as Han (2490; 82.2%) and Zhuang (373, 12.3%). Similarly, most patients reported their race as Han (1693; 80.3%) and Zhuang (254, 12.0%). Most caregivers (1289, 42.5%) had completed junior high school, followed by those with a high school education (748, 24.7%). Caregivers generally had higher education levels than the patients they cared for (*P*-value < 0.001). 33.4% (1012) caregivers and 42.7% (901) patients indicated that they had used traditional medicine. Respondents represented all age groups and came from diverse inpatient settings. Most respondents were from the internal medicine discipline (caregivers: 778, 25.7%; patients: 576, 27.3%) and surgical disciplines (caregivers: 618, 20.4%; patients: 476, 22.6%).

**Table 1:** Characteristics of caregivers and their cared inpatients

	Caregivers, No. of total (%)	Inpatients, No. of total (%)	*P*-values
Total No. of respondents	3030	2108	
Age, mean(SD), y	44.0 (11.0)	49.7 (17.1)	< 0.001
Sex			0.442
Female	1603 (52.9)	1139 (54.0)	
Male	1427 (47.1)	969 (46.0)	
Race			0.010
Zhuang	373 (12.3)	254 (12.0)	
Yao	102 (3.4)	86 (4.1)	
Han	2490 (82.2)	1693 (80.3)	
Others^a^	65 (2.1)	75 (3.6)	
Education			< 0.001
Elementary school and below	408 (13.5)	755 (35.8)	
Junior high school	1289 (42.5)	786 (37.3)	
High school	748 (24.7)	364 (17.3)	
College and above	585 (19.3)	203 (9.6)	
Relationship with patients			
Child	1235 (40.8)	–	
Non-child relative	1743 (57.5)	–	
Non-relative	52 (1.7)	–	
(Cared) patient disciplines			0.154
Internal medicine	778 (25.7)	576 (27.3)	
Surgery	618 (20.4)	476 (22.6)	
Cardiovascular and thoracic surgery	270 (8.9)	186 (8.8)	
Obstetrics and pediatrics	456 (15.0)	290 (13.8)	
Orthopedics	114 (3.8)	80 (3.8)	
Neuroscience	152 (5.0)	109 (5.2)	
Critical care and integrated medicine	177 (5.8)	131 (6.2)	
Otolaryngology	246 (8.1)	167 (7.9)	
Comprehensive^b^	175 (5.8)	88 (4.2)	
Missing	44 (1.5)	5 (0.2)	
Used traditional Chinese medicine			< 0.001
Yes	1012 (33.4)	901 (42.7)	
No	2018 (66.6)	1207 (57.3)	
Discoformt between Western and traditional Chinese medicine			0.316
Yes (If you select "No," please proceed to the following)	851 (28.1)	620 (29.4)	
No	2179 (71.9)	1488 (70.6)	
Methods to mitigate discoformt (Multiple choices)			
Seek help from family and friends	558 (65.6)	443 (71.5)	0.022
Consult professional psychologists or other health professionals	628 (73.8)	460 (74.2)	0.346
Self-manage	399 (46.9)	303 (48.9)	0.219

^a^ Others refer to Miao, Dong, Mulao and other ethnic groups in Guangxi

^b^ "Comprehensive" category includes traditional medicine, rehabilitation, day treatment, and day radiotherapy

Caregivers’ perspectives on digital healthcare tools are summarized in Table 2. While 2640 (87.1%) reported having used digital healthcare tools in their care practice, most of them (81.1%) thought these tools had either not or only partially addressed their needs and skill training. Care partners expressed a strong desire for digital healthcare systems providing information on medical regulations and insurance (1975, 65.2%), guidance on patient visits and personalized care (1878,62.0%), and mental health support and assistance methods (1754, 57.9%). 851(28.1%) caregivers experience discomfort or difficulty due to the conflict between Western and traditional Chinese medicine. When faced with this conflict, care partners collectively prioritized seeking help through mental health professionals or related experts, family and friends, and self-management strategies.

**Table 2:** Caregivers’ perspective on digital healthcare tools

Survey item	Caregivers, No. of Total (%)	Patients, No. of Total (%)	P-values
Have you ever used digital healthcare care tools^a^?			< 0.001
Yes (If you select "Yes," please proceed to the following question)	2640 (87.1)	1308 (62.0)	
No	390 (12.9)	800 (38.0)	
Do you think the current digital healthcare care system takes into account the care needs and skills training of caregivers?			
Fully considered	458 (17.3)	–	
Partially considered	1948 (73.8)	–	
Not considered	234 (8.9)	–	
What content would you like the digital healthcare care system to provide to help caregivers understand care needs and training skills (Muliple choices)?			
Knowledge and skills in disease management and first aid	1557(51.4)	–	
Mental health support and assistance methods	1754 (57.9)	–	
Information on medical regulations and insurance	1975(65.2)	–	
Guidance on patient visits and personalized care	1878 (62.0)	–	
Multilingual multimedia support^b^	1625 (53.6)	–	
Remote health monitoring	931 (30.7)	–	
What cultural background factors do you value most in using digital healthcare care tools (Muliple choices)?			
Language diversity	1720 (56.8)	1104 (52.4)	0.002
Integration of traditional Chinese medicine knowledge	2031 (67.0)	1349 (64.0)	0.025
Folk customs and beliefs	1440 (47.5)	1012 (48.0)	0.733
Local medical resource and distribution availability	1340 (44.2)	1016 (48.2)	0.005
What content could help increase your acceptance of digital healthcare tools (Muliple choices)?			
Dialect support	1487 (49.1)	1131 (53.7)	0.001
Integration of traditional Chinese medicine knowledge	1894 (62.5)	1372 (65.1)	0.058
Adding interactive health management tools (eg., health monitoring and medication reminders)	2222 (73.3)	1407 (66.7)	< 0.001
Mental health support	1046 (34.5)	686 (32.5)	0.134
What approaches could help increase your access to digital healthcare tools (Muliple choices)?			
Conducting training and promotional activities	1763 (58.2)	1248 (59.2)	0.466
Colloborating with local hospitals/health clinics	2065 (68.2)	1398 (66.3)	0.169
Designing user-friendly interfaces	2193 (72.4)	1545 (73.3)	0.468
Establishing user participation and feedback mechanisms	1855 (61.2)	1159 (55.0)	< 0.001
Providing social interaction features^c^	1005 (33.2)	685 (32.5)	0.613

^a^ Defined for participants as follows: digital healthcare tools refer to disease management-related digital tools (eg, mobile health monitoring apps, mini-programs, software, websites, WeChat public accounts)

^b^ Support includes multilingual websites, virtual assistants, animated films, and information

^c^ Defined for participants as follows: social interaction features refers to establish group chats for easy communication

The survey instrument inquired about caregivers’ emphasis on integrating cultural factors into digital healthcare tools. Out of the four cultural background factors assessed—language diversity, traditional medicine, and folk customs—2,031 (67.0%) participants expressed that they prioritize traditional Chinese medicine practices the most. Regarding accepting digital healthcare tools, 2,222 (73.3%) indicated they would be more inclined to embrace digital healthcare products that offered interactive health management features (e.g., health monitoring and medication reminders). Finally, a majority of caregiver respondents believed that providing user-friendly interfaces (2,193, 72.4%) and partnering with local hospitals/health clinics (2,065, 68.2%) would improve their access to digital healthcare tools.

**Fig. 1:**
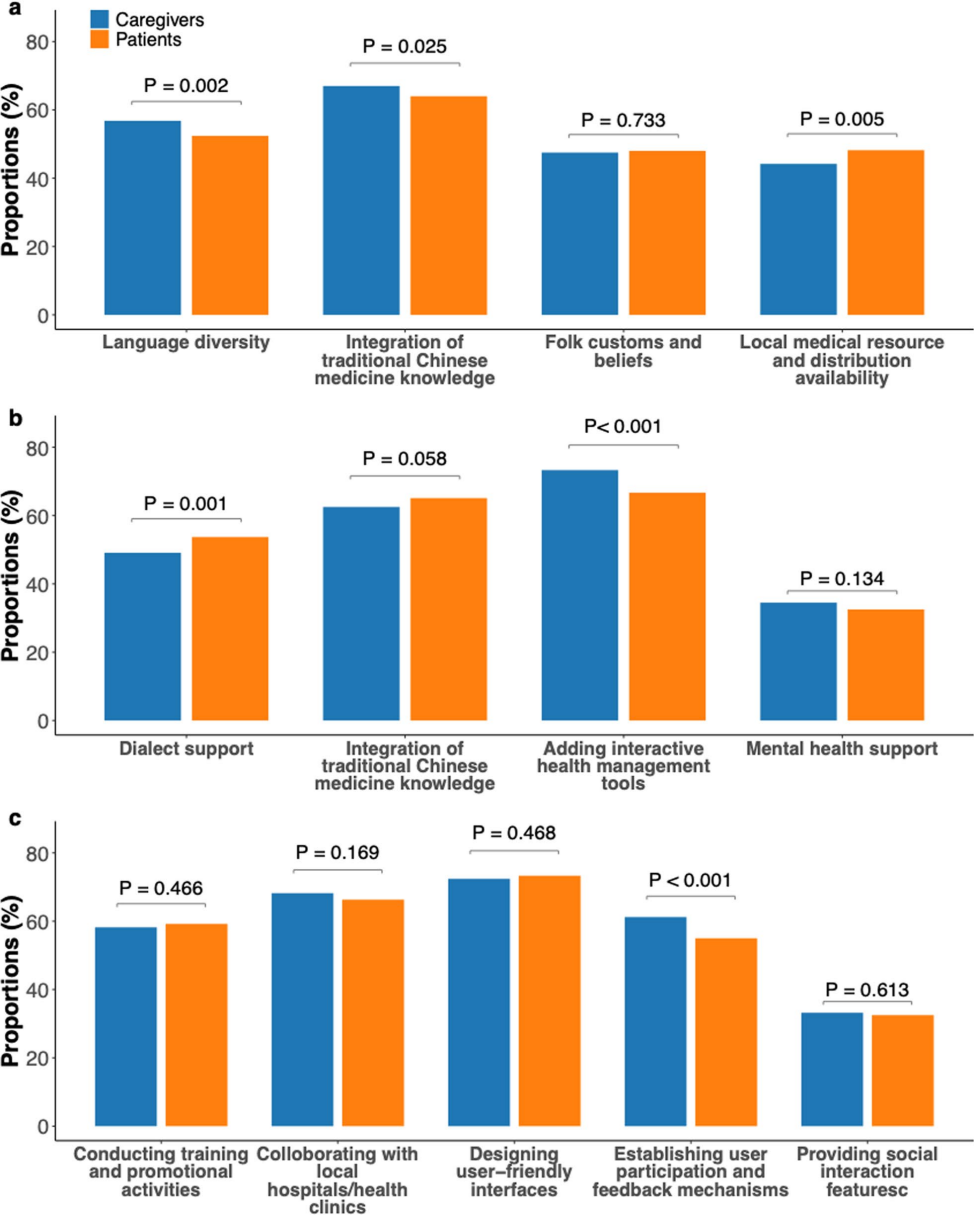
Caregiver versus patient perspectives on cultural adaptation for digital health tools. **a**, valued cultural factors in tool design. **b**, preferred content for increasing tool acceptance. **c**, preferred approaches for improving access

Compared to caregivers, patients indicated a lower experience of using digital healthcare tools (1308, 62.0%; *P*-value < 0.001), which may be linked to their lower education levels (Fig. 1). Patients shared similar preferences with caregivers on prioritizing incorporating cultural factors, with traditional Chinese medicine practices being the most valued. Furthermore, patients placed less emphasis on the inclusion of interactive health management tools to increase acceptance (1407, 66.7%; *P*-value < 0.001) and were also less inclined than caregivers to value user feedback mechanisms for improving access (1159, 55.0%; *P*-value < 0.001).

## Discussion

This study reveals that caregivers prioritize the integration of traditional medicine practices and interactive health management features in digital tools more highly than patients, offering a crucial directive for digital health developers. To translate this into practice, traditional medicine integration could encompass features such as symptom checkers that blend Traditional Chinese Medicine diagnostics with Western medical assessments, alerts for herb-drug interactions, or virtual consultations with practitioners holding dual licenses in both medical paradigms. Furthermore, caregiver-specific functionalities should prioritize feedback mechanisms, such as AI-driven care coordination dashboards, to mitigate burnout associated with complex care management [9, 10].

These findings underscore an imperative for digital health: bridging biomedical and cultural care paradigms to retain relevance in regions where traditional medicine is foundational (e.g., Guangxi in China, South Asia, Sub-Saharan Africa, and Indigenous communities) [2, 8]. Addressing this necessitates a co-design framework involving caregivers, traditional healers, and local communities. Prototype hybrid care modules could align traditional medicine practices with modern disease ontologies, advancing the WHO traditional medicine goals while meeting the United Nations’ Sustainable Development Goals for equitable innovation [6, 7].

Study limitations include potential self-reporting bias; future research should triangulate preferences with behavioral data and extend to underrepresented regions where cultural norms may differentially impact digital adoption.

## Conclusions

Digital health tools require user-centered designs that proactively incorporate traditional medicine practices and prioritize caregiver-specific functionalities—a strategy that could enhance global accessibility and mitigate disparities in culturally diverse populations.

## Data Availability

The datasets used and/or analyzed in this study are not publicly accessible due to participant privacy concerns, but they can be obtained from the corresponding author upon reasonable request.
